# The long-term outcomes of the Anderson-Kestenbaum procedure

**DOI:** 10.3389/fopht.2023.1247385

**Published:** 2023-09-29

**Authors:** Jeffrey Kuziel, Hannah Pope, Aishwarya J. Kothapalli, Scott A. Larson, Arlene Drack, Alina V. Dumitrescu

**Affiliations:** Department of Ophthalmology and Visual Sciences, The University of Iowa, Iowa City, IA, United States

**Keywords:** Anderson Kestenbaum procedure, nystagmus, head position, long term outcome, surgery

## Abstract

**Introduction:**

Nystagmus is an involuntary, conjugated, rhythmic movement of the eye that can be idiopathic or secondary to ocular or neurologic pathologies. Patients with nystagmus often have a position of gaze in which their symptoms are dampened or absent, referred to as the “null zone.” The Anderson-Kestenbaum procedure is a bilateral recess-resect procedure of the four horizontal rectus muscles which aims to bring the null position into the primary gaze. This study aims to further elucidate long-term outcomes and factors associated with optimal postoperative outcomes.

**Methods:**

Patients with a diagnosis of nystagmus and a surgical code for strabismus between June 1990 and August 2017 were considered for inclusion in the study. Patients were included if they had undergone the Anderson-Kestenbaum procedure and had follow-up lasting at least 24 months post-operatively. Data collected included demographic information, characteristics of the nystagmus, underlying etiology of nystagmus, and pre-and post-operative measurements.

**Results:**

25 patients were included. At their last recorded follow-up, 44% of patients achieved an optimal surgical outcome -an abnormal head position of 10 degrees or less. 88% of patients showed an overall improvement in their head posture at the last follow-up. The absence of an abnormal head position at the visit closest to 24 months post-operatively was found to be significantly associated with the lack of a significant head position at the last follow-up visit. Optimal surgical outcomes were not significantly associated with the underlying diagnosis, the direction of the abnormal head position, or the type of nystagmus.

**Discussion:**

The relatively long follow-up of this cohort allows this study to further elucidate the long-term outcomes of the Anderson-Kestenbaum procedure. Overall, our results suggest that although improvement in head position post-operatively is likely, it is still expected that many patients will have a residual abnormal head position after the procedure. The results of this study are helpful in counseling patients, especially knowing that if they do not have a significant head position at 24 months follow-up, they are unlikely to develop one. However, due to the small sample size, larger cohorts and more standardized follow-up may provide further insight into the procedure’s outcomes.

## Introduction

Nystagmus is an involuntary, conjugated, rhythmic movement of the eye. This occurs along the horizontal, vertical, or torsional axis, or a combination of these (1). Nystagmus, present at age 6 months or less, is termed “infantile nystagmus” (2). Infantile Nystagmus can be idiopathic or secondary to ocular or neurologic pathologies, including albinism, retinal disease, early visual deprivation, congenital malformations, or periventricular leukomalacia. Ultimately, nystagmus can negatively impact visual acuity, leading to instability of images on the retina (1).

Patients with nystagmus will often adopt a position of gaze in which oscillation of the eyes will be dampened or absent. This is often referred to as the “null zone” and is the position of gaze in which patients have their best visual acuity (3, 4). Certain patients will adopt an abnormal compensatory head posture to achieve this null position and minimize the effects of nystagmus. This posture can be a head turn along the horizontal or vertical axis, a head tilting, or a combination of these positions. If uncorrected, this abnormal head posture can lead to neck and spine issues, impairment of social interactions, and decreased quality of life (5).

Between 1953 and 1954, Anderson, Kestenbaum, and Goto each independently proposed the use of eye muscle surgery to improve abnormal head posture associated with Nystagmus (6–8). The Anderson-Kestenbaum procedure (AKP) is a bilateral recess-resect procedure of the medial and lateral rectus muscles, which has since been modified to further correct abnormal head posture (9). In 1973, Parks proposed measurements of 5 mm and 6 mm of recession/resection of the medial rectus muscles and 7 and 8 mm on the lateral rectus muscles (10). Later, Calhoun and Harley proposed 40% augmentation, while Nelson and colleagues proposed a 60% augmentation to further improve outcomes for more significant abnormal head positions (11, 12). Today, including these modifications, the AKP is a common procedure to correct various degrees of nystagmus-associated abnormal head posture. The AKP brings the null position into the primary gaze. In doing so, it aims to improve eye contact, relieve neck problems, and broaden the null zone of the nystagmus (2, 13). Some studies have also shown it improves visual acuity and reaction time (14).

Although the procedure is effective for many in the short term, patients may require additional surgery years later for new or recurring abnormal head postures (3, 15). Our study aims to further elucidate the long-term outcomes of patients undergoing the AKP for nystagmus-associated abnormal head posture and factors that may be associated with a more favorable surgical outcome.

## Methods

### Patient selection

Patients diagnosed with nystagmus who were seen from June 1990 to August 2017 (n=3981) were cross-referenced with patients who had a recorded surgical procedure for strabismus during the same period of time (n=7779). 324 charts contained both a diagnostic code for nystagmus and a surgical code for a strabismus procedure during this timeframe.

Inclusion criteria required that patients had nystagmus and abnormal (compensatory) head posture for null positioning. It also required that patients underwent the bilateral recess/resect procedure with Parks’ 5-6-7-8 measurements for small head positions or with 40 or 60% augmentations for larger head positions. Patients with concomitant strabismus were included as well. It was recorded if the correction of their strabismic deviation was included in the surgical plan. Patients were also required to have at least 24 months of follow up post-operatively and have pre- and post-operative measurements available.

Patients were excluded if their nystagmus was acquired. They were also excluded if they underwent extraocular muscle (EOM) surgery solely for strabismus or abnormal head posture related to a previous strabismus surgery not associated with null positioning (n=179). Patients were also excluded if they underwent an EOM procedure for abnormal head posture that was not an AKP (n=64). Additional exclusion criteria included patients with abnormal head position not due to nystagmus (e.g., monocular elevation deficiency or superior oblique palsy) (n=21) or those who underwent additional EOM surgery for either abnormal head position or strabismus prior to the AKP (n=16). These procedures were strabismus surgery, Anderson procedures alone, or tenotomies.

Overall, 25 patients met the eligibility criteria for inclusion in the study.

### Data collection

Data collected from patient charts included demographic information as well as characteristics of the nystagmus. The etiologies of nystagmus included oculocutaneous albinism (OCA), optic nerve hypoplasia, congenital stationary night blindness (CSNB) and idiopathic infantile nystagmus (INS). The INS category included all patients that were diagnosed with congenital nystagmus by the examining physician and did not have other underlying conditions that were known to be contributing to their nystagmus with or without a complete workup.

Patients were also documented as to whether they had undergone a complete nystagmus workup consisting of all the necessary tests to diagnose an underlying condition presenting with congenital nystagmus.

Visual acuity was recorded pre-operatively, at the second follow up (4 to 6 weeks postoperatively), and at the patient’s last documented follow-up visit. Due to the young age of many patients, visual acuity could not be measured. Instead, the central, unsteady, maintained (CUSM) method was used to assess fixation in these patients.

The head position recorded at the pre-operative appointment was designated as the presurgical head position. Head position was categorized as horizontal, vertical, or combination based on the axis of the abnormal head position. Combination head positions were defined as those that occurred along multiple axes. Due to inconsistencies in documenting the exact degrees of head turn, head positions were categorized as small, medium, or large. The designation as small, medium, or large for the head position refers to less than 30 degrees for small, 30 to 45 degrees for medium, and more than 45 degrees for large. The preoperative notes and the operative plans correlated, and the procedure of choice was Parks’ original 5-6-7-8 recess-resect procedure for the small head position, 40% augmentation for the medium, and 60% augmentation for the large head position.

At the follow-up visit closest to 24 months and at the last documented follow-up visit, it was evaluated if patients had improved their abnormal head position. At the last documented visit, it was also noted if patients had an optimal head position. An optimal head position was defined as an abnormal head posture that was either absent or ≤10 degrees. Patients were designated as still having a significant head position if their abnormal head posture remained greater than 10 degrees at the time of follow up.

### Data analysis

Collected Data was compiled into a database using FileMaker Pro. Fischer’s exact tests in R were performed for the categorical variables assessing both improvements in head position and optimal outcomes and t-tests in R were used for continuous variables.

## Results

25 charts met the inclusion criteria with a mean follow-up time of 102 months (Min. 26 months, Max. 204 months). At baseline, our cohort included nearly equal numbers of male (48%) and female (52%) patients. The majority of patients (76%) had INS, while 24% had an underlying inherited eye disorder. 52% of patients had undergone a complete workup for nystagmus, including electrophysiology, magnetic resonance imaging, and optic coherence tomography. Previous studies have shown the importance of complete eye examinations as well as these testing modalities in determining the etiology of nystagmus (16). It is possible that more patients would have been diagnosed with an underlying condition if they underwent complete workup. The horizontal axis was the most common direction of the abnormal head position (68%), and most head positions were classified as medium in size (64%).The complete baseline characteristics of the cohort are displayed in **Table 1**.

**Table 1:** Cohort description (n=25).

**Sex**	Male: 48% (12/25)	Female 52% (13/25)	
**Age at Time of Surgery**	Min: 19 months	Max: 153 months	Avg: 66 months (SD 36.9)
**Underlying Diagnosis**	Inherited Eye Disorder: 24% (6/25)	Idiopathic Congenital Nystagmus: 76% (19/25)	
**Associated Pre-Operative Strabismus**	Yes: 48% (12/25)	No: 52% (13/25)	
**AHP Axis**	Horizontal: 68% (17/25)	Combined: 20% (5/25)	Vertical: 12% (3/25)
**Size of AHP**	Small: 20% (5/25)	Medium: 64% (16/25)	Large: 16% (4/25)
**Length of Follow Up**	Min: 26 months	Max: 204 Months	Avg: 102 months (SD 47.8)

Overall, 44% of patients achieved the primary outcome of an optimal outcome (defined as an abnormal head position of 10 degrees or less) at their last recorded follow-up visit, while 56% remained with a significant abnormal head position. A significant abnormal head position is defined as any head position which is greater than 10 degrees. 88% of patients had an overall improvement in their abnormal head position from their pre-operative appointment to their last documented follow-up (**Table 2**). An improved head position describes any documented improvement in head position, regardless of the magnitude of change. 4 patients did not have any appointments that were close to 24 months after their operation (defined as a post-operative visit between 20 and 31 months); however, they were noted to have a significant abnormal head position at follow-up visits around 12 months and around 36 months postoperatively. Thus, we assumed that these patients also had an abnormal head position 24 months after their operation, which would have been noted had they presented for follow-up at that time.

**Table 2:** Overall abnormal head position results at last follow up.

**Improvement in AHP**	88%
**Significant AHP at Last Follow Up**	56%
**Optimal Outcome at Last Follow Up**	44%
**Significant AHP at 24 Months**	68%
**Optimal outcome at 24 Months**	32%

**Figure 1:**
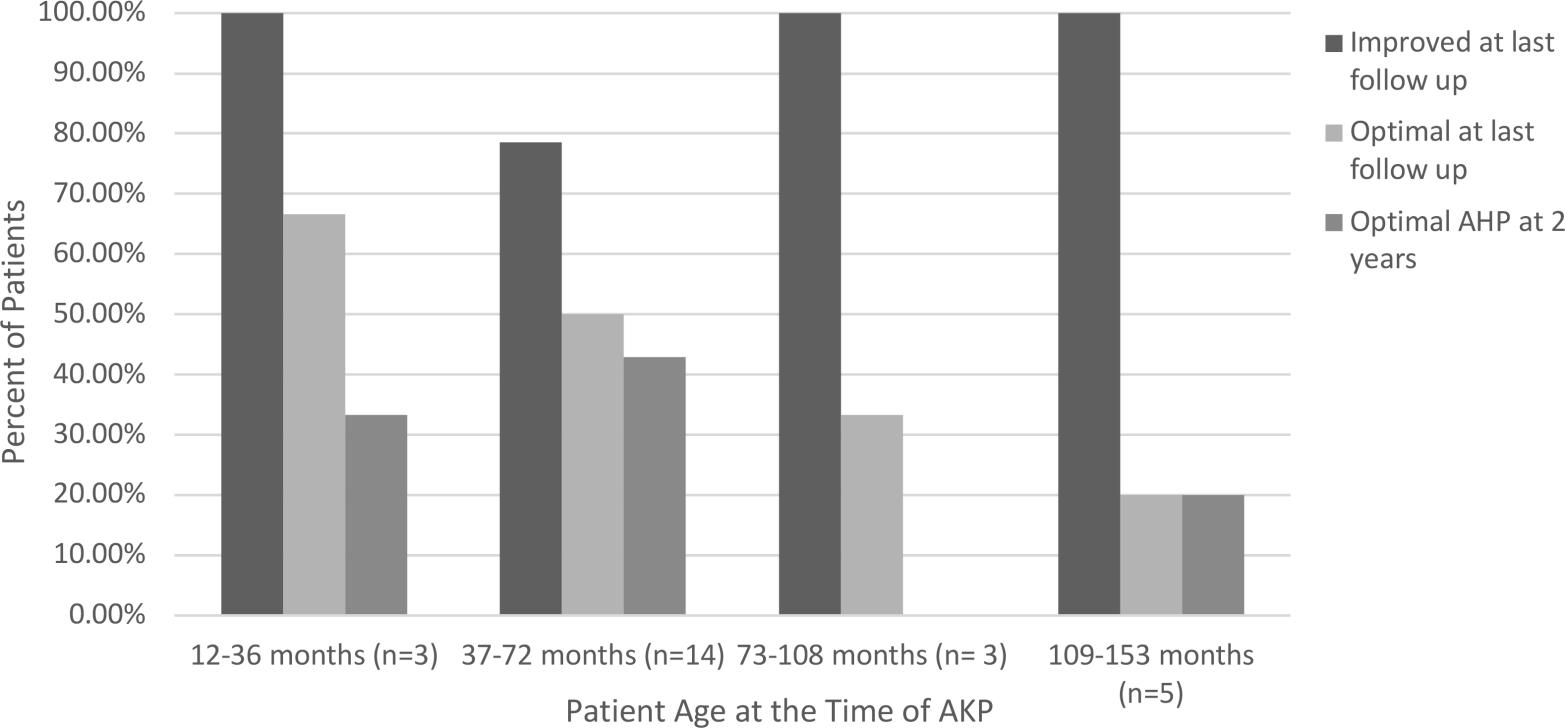
Age vs. Head Position at 2-Year and Last Follow Up.

Several variables were analyzed to see whether they were associated with optimal outcomes after AKP. These variables included the gender of the patient, the presence of a known underlying diagnosis, associated strabismus, the direction of the abnormal head position, the type of nystagmus, and the patient’s age at the time of surgery and at their last follow up appointment (**Table 3**). None of these variables demonstrated statistical significance; however, the absence of a significant head position at the visit closest to 24 months was significantly associated with the lack of a significant head position at their last follow-up visit (OR 23, p=0.0072) (**Table 4**). Head position characteristics and outcomes for each patient included in the analysis are listed in **Table 5**. Although the association was not statistically significant, higher rates of optimal outcomes were seen in patients who underwent AKP at a younger age (**Figure 1**). Patients with motor nystagmus had a higher success rate (optimal outcome) than patients with a known underlying inherited eye disorder in this cohort. Both groups had similar rates of improvement in their head posture after the surgery (**Table 3**). The majority of patients in this cohort had a horizontal head posture. The rate of success and improvement were comparable between horizontal, vertical, and combined head positions. (**Table 3**).

**Table 3:** Head posture results at last follow up.

Sex		
Male	Improved 92% (11/12)	Optimal 50% (6/12)
Female	Improved 85% (11/13)	Optimal 38% (5/13)
Underlying Diagnosis		
Inherited Eye Disorder	Improved 100% (6/6)	Optimal 17% (1/6)
Congenital Nystagmus	Improved 84% (16/19)	Optimal 53% (10/19)
Associated Pre-Operative Strabismus		
Yes	Improved 83% (10/12)	Optimal 33% (4/12)
No	Improved 92% (12/13)	Optimal 38% (5/13)
AHP Axis		
Horizontal	Improved 88% (15/17)	Optimal 47% (8/17)
Vertical	Improved 100% (3/3)	Optimal 33% (1/3)
Combined	Improved 80% (4/5)	Optimal 40% (2/5)
Size of AHP		
Small	Improved 60% (3/5)	Optimal 60% (3/5)
Medium	Improved 94% (15/16)	Optimal 44% (7/16)
Large	Improved 100% (4/4)	Optimal 25% (1/4)

**Table 4:** Significant AHP at 24 months vs. last follow up.

	Significant AHP at Last Follow Up				
Significant AHP at 24 Months Follow Up?	No	Yes	Total	p-value	OR
No	7	1	8	0.0072	23
Yes	4	13	17		
Total	11	14	25		

**Table 5:** Patient head position characteristics and outcomes.

Patient	Size of AHP	Direction of AHP	Significant AHP at 2 years follow-up?	Significant AHP at last follow up?
1	Medium	Horizontal	Yes	Yes
2	Medium	Combined	Yes	Yes
3	Medium	Horizontal	Yes	Yes
4	Large	Horizontal	Yes	Yes
5	Medium	Horizontal	No	No
6	Medium	Horizontal	Yes	Yes
7	Medium	Horizontal	No	No
8	Medium	Combined	No	No
9	Small	Combined	Yes	No
10	Small	Combined	Yes	Yes
11	Medium	Vertical	No	Yes
12	Medium	Horizontal	No	No
13	Medium	Horizontal	No	No
14	Medium	Horizontal	Yes	Yes
15	Small	Vertical	Yes	No
16	Medium	Horizontal	Yes	No
17	Small	Horizontal	No	No
18	Medium	Vertical	Yes	Yes
19	Large	Horizontal	No	No
20	Medium	Combined	Yes	Yes
21	Medium	Horizontal	Yes	No
22	Small	Horizontal	Yes	Yes
23	Large	Horizontal	Yes	Yes
24	Large	Horizontal	Yes	Yes
25	Medium	Horizontal	Yes	Yes

Of the 12 patients who had associated strabismus pre-operatively (with correction included in the surgical plan), 50% had overcorrection of their strabismus, 17% had a resolution, and 33% were undercorrected. Of the 13 patients who did not have pre-operative strabismus, 31% developed a new strabismus after undergoing AKP.

Patients were also assessed for changes in visual acuity following AKP. Overall, 40% of patients out of the cohort demonstrated some improved visual acuity post-operatively, 20% had a worsening visual acuity, and 20% had an unchanged visual acuity. 20% were unable to be compared due to the use of the CUSM system pre-operatively and Snellen visual acuity post-operatively. Patients who demonstrated improvement in their visual acuity were younger at the time of surgery (between 3 and 9 years of age), and only 3 of the 10 patients in this group had a known diagnosis of an inherited eye disorder. Patients who demonstrated worsening in their visual acuity were between 3 and 12 years of age at the time of their surgery, and 3 of the 5 patients in this group had a known diagnosis of an inherited eye disorder.

## Discussion

To date, several studies have investigated the success of various iterations of the AKP in correcting abnormal head position due to infantile nystagmus. These studies have shown different degrees of optimal outcomes and varied in mean follow-up time from 13 to 54 months (11, 12, 17–23). In 2020, Zheng and colleagues reported on the longer-term outcomes of a cohort of patients who underwent surgery to correct nystagmus-related abnormal head posture. They reported success (abnormal head position ≤10°) in 93% of patients who were seen for follow up at 10 years (n=14). However, their cohort included broader criteria for inclusion than our study, including patients with acquired nystagmus and those who underwent nonstandard procedures (24).

Given the relatively long follow-up of our cohort, we were able to elucidate the long-term procedural success of the AKP. Our study demonstrated that long-term improvement in head position with the AKP was likely and achieved in most of our patients. Although improvement in head position was noted in most patients, over half of our patients were still with a significantly abnormal head position at their last follow-up visit. This finding is one of many in our study that may be relevant to counseling patients in a clinical setting. Other implications of our study for the counseling of patients include the rates of other post-operative changes, including the development of strabismus and change in visual acuity.

Regarding changes in visual acuity, it is possible that increased visual acuity post-operatively could be related to patients getting older and a reflection of their maturation. We cannot prove that this is related to an improvement in abnormal head posture post-operatively. Some of the patients who had worsening of visual acuity after surgery also have an underlying genetic disorder. It is possible that the worsening of visual acuity may be related to a worsening of underlying conditions rather than as a consequence of the AKP. Some patients were initially tested with LEA symbols and later as they got older they were tested with Snellen symbols. Since they are not exactly equivalent, it may be just an appearance of worsening in visual acuity.

Additionally, our study demonstrated that it could be difficult to initially predict who will have an optimal outcome after an AKP. Factors that we were able to measure pre-operatively such as sex of the patient and underlying diagnosis were not significantly associated with optimal outcomes. However, it is useful to know that if patients do not have an abnormal head position at 24 months after their operation, it is very unlikely that they will develop one by their last followup visit.

The overall small sample size limits our study. In attempting to control for several confounding variables, the sample size became much smaller than anticipated. While we were unable to identify pre-operative factors that would best predict long-term success with the AKP, future studies with larger sample sizes may be able to demonstrate significant correlations. Additionally, while abnormal head position at 24 months was associated with a head position at the patient’s last follow-up, the 24-month appointment is an arbitrary time point. It may be beneficial in future studies to perform a prospective study with standardized follow-up to determine if follow-up closer to the procedure would be associated with optimal long-term outcomes.

## Data availability statement

The raw data supporting the conclusions of this article will be made available by the authors, without undue reservation.

## Ethics statement

The studies involving humans were approved by University of Iowa institutional review board. The studies were conducted in accordance with the local legislation and institutional requirements. Written informed consent for participation was not required from the participants or the participants’ legal guardians/next of kin because this is a retrospective chart review and individual consent was waived.

## Author contributions

JK, HP, AK, SL, ADr, and ADu, All author’s contributed to the study design, data collection, data analysis, manuscript writing, or critical review. All authors contributed to the article and approved the submitted version.
